# Dysregulated Phosphate Metabolism, Periodontal Disease, and Cancer: Possible Global Health Implications

**DOI:** 10.3390/dj7010018

**Published:** 2019-02-11

**Authors:** Ronald B. Brown

**Affiliations:** School of Public Health and Health Systems, University of Waterloo, Waterloo, ON N2L 3G1, Canada; r26brown@uwaterloo.ca; Tel.: +1-519-578-2094

**Keywords:** phosphate toxicity, periodontal disease, cancer, tumorigenesis, chronic kidney disease-mineral and bone disorder, dental calculus, resorption, RNA, global health, ectopic calcification

## Abstract

An association between periodontal disease and cancer has been established in recent studies, but no common etiology has been identified in the hopes of reducing the global burden of these non-communicable diseases. This perspective article hypothesizes that the determinant mediating the association of periodontal disease with cancer is dysregulated phosphate metabolism. Phosphate, an essential dietary micronutrient, is dysregulated in chronic kidney disease, and both cancer and periodontal disease are associated with chronic kidney disease. Reviewed evidence includes the association between phosphate toxicity and cancer development, and the association between periodontal disease and chronic kidney disease-mineral and bone disorder includes conditions such as ectopic calcification and bone resorption, which may be indirectly related to periodontal disease. Dental calculus in periodontal disease contains calcium phosphate crystals that are deposited from excess calcium and phosphate in saliva. Alveolar bone resorption may be linked systemically to release of parathyroid hormone in response to hypocalcemia induced by hyperphosphatemia. More research is needed to examine the role of dysregulated phosphate metabolism in periodontal disease.

## 1. Introduction

The global burden of periodontal disease, which accounts for the majority of the 442 billion USD spent on oral disease costs, share risk factors, and social determinants with other non-communicable diseases such as diabetes, heart disease, and cancer [1]. Recent studies investigating the link between periodontal disease and various cancers include meta-analyses of periodontal disease and cancer [2,3], studies of periodontal disease and cancer in postmenopausal women [4,5], in male non-smokers [6], and studies and meta-analyses of periodontal disease linked to specific cancers including breast cancer [7,8], pancreatic cancer [9,10], oral cancer [11,12], colorectal cancer [13,14], lymphoma [14,15], head and neck cancer [16], lung cancer [17,18], prostate cancer [19], and gastric cancer [20]. To the author’s knowledge, no findings from major studies published within the last five years have failed to support an association of periodontal disease with at least some type of cancer risk. Discrepancies between studies regarding specific cancers may be accounted for by the study design, population studied, and method used to measure periodontal disease [5].

Despite consistent findings supporting a positive association between periodontal disease and cancer, replete with hypotheses implicating oral microorganisms and systemic inflammation, a common causal mechanism has not been identified in the etiology of these two non-communicable diseases. It is not clear if periodontal disease causes cancer, or cancer causes periodontal disease, or some other common pathophysiological determinant causes both diseases. This perspective article reviews evidence supporting a novel hypothesis that the association of periodontal disease with cancer may be mediated by the pathophysiological determinant of dysregulated phosphate metabolism. The reader should bear in mind that statements related to the proposed hypothesis in this article are hypothetical, and further research is required for acceptance or rejection of this hypothesis. The discovery of a common cause linking periodontal disease and cancer has possible global health implications, suggesting a reduction in the global burden of these non-communicable diseases by modifying their common lifestyle risk factors and determinants.

## 2. Phosphate Toxicity as a Global Health Burden

Phosphorus, an essential dietary micronutrient, is ingested as phosphate in food and food additives. The highest amount of phosphate within the body is stored in bone as calcium phosphate. Levels of serum phosphate are regulated through a hormonal network, involving the kidneys, parathyroid glands, intestines, and the skeletal system [21]. Bioactive vitamin D, 1,25(OH)_2_D_3_, increases intestinal absorption of dietary phosphate, mainly through enhanced expression of sodium-phosphate 2b cotransporters. Fibroblast growth factor 23 (FGF23) is produced within osteocytes and osteoblasts of bone. Working in conjunction with its co-factor, Klotho, FGF23 lowers serum phosphate levels, and increases urinary phosphate excretion by suppressing reabsorption through action of sodium-phosphate cotransporters in the kidneys. Phosphorus renal reabsorption is also decreased by parathyroid hormone (PTH). If phosphate is dysregulated due to kidney burden and excessive dietary phosphate intake, serum phosphate levels may rise (hyperphosphatemia) and excessive phosphate may be sequestered in cellular tissue producing a pathological condition called phosphate toxicity. 

Phosphate toxicity is emerging as a global health concern as average amounts of dietary phosphate intake increase to approximately double the Recommended Dietary Allowance of 700 mg per day for an adult [22]. An excessive amount of phosphate stored in the body tissue may not always correlate with serum levels. It is possible that phosphate toxicity may be present in the body cells even in normophosphatemia, disturbing the function of almost every system in the body, including the muscular, skeletal, and vascular systems, and increasing morbidity and mortality [23]. Genetic evidence from animal experiments shows that phosphate toxicity may also accelerate mammalian aging [24], and phosphate toxicity is associated with tumorigenesis [25].

## 3. Dysregulated Phosphate Metabolism and Cancer

Evidence supporting the role of dysregulated phosphate metabolism and phosphate toxicity in tumorigenesis has been detailed elsewhere [26,27]; a very brief summary of that evidence with important relevance to periodontal disease is presented here. Cancer cells express more phosphate cotransporters within their cell membranes than normal cells [28], which allow cancer cells to absorb and retain greater amounts of phosphate from the tumor microenvironment. Solid tumors have filopodia and lamellipodia that extend cancer cell membranes throughout the tumor microenvironment [29]. Phosphorus is a limiting factor in biological growth [30] and is the least abundantly supplied element in the formation of nucleic acids DNA and RNA. The sequestration of dysregulated amounts of phosphorus in cancer cells is associated with additional biosynthesis of ribosomal RNA [31], which increases protein synthesis necessary for cancer cell growth and tumorigenesis. Of relevance, detection of the overexpression of circulating microRNA fragments associated with dysregulated RNA biogenesis has potential as a cancer biomarker [32].

While serum phosphorus levels are not always a reliable indicator of phosphate stored in the body, a study of cancer patients found that they had abnormally higher serum phosphate levels compared to control patients [33]. The Health Professionals Follow-Up Study found that high-grade prostate cancer was associated with high dietary phosphate levels [34]. High dietary phosphorus fed to experimental animals caused skin cancer [35] and lung tumors [36]. Of relevance, experimental studies showing causative effects of cancer from feeding the milk protein casein [37] may not have controlled for high levels of phosphorus within casein, which is classified as a phosphoprotein [38]. Other studies have shown that high amounts of phosphate stimulate tumor neovascularization [39] and cell signaling in cancer growth [40], and are associated with chromosome instability [41] and metastasis [42]. Phosphate toxicity also contributes to systemic inflammation and malnutrition [43], which is seen in terminally ill cancer patients with cachexia.

## 4. Periodontal Disease

The periodontium functions to connect teeth to bone; its structures consist of the cementum, periodontal ligament, gingiva, and alveolar bone. Periodontal disease is the most common cause of adult tooth loss [44]. Many mediators of inflammation in periodontal disease are associated with cancer risk, such as C-reactive protein (CRP), Matrix metalloprotenases (MMP), Tumor Necrosis Factor (TNF), and Interluekin (IL) [45]. Inflammation within the periodontium may begin as gingivitis and eventually progress to periodontitis, which is usually associated with the accumulation of dental plaque or calculus. Dental calculus is formed supragingvally and subgingivally when biofilms rich in bacteria are mineralized with various crystals of calcium phosphate, including octacalcium phosphate = Ca_4_H(PO_4_)_3_ · 2H_2_O, brushite = CaH(PO_4_) · 2H_2_O, hydroxyapatite = Ca_5_(PO_4_)_3_(OH), and whitlockite that contains a small amount of magnesium and other elements = β-Ca_3_(PO_4_)_2_ [46]. While growth of bacteria in the oral microbiota is associated with periodontal disease, there is currently insufficient evidence to either support or exclude a causative role of bacterial invasion in the etiology of periodontal disease [47]. 

**Figure 1:**
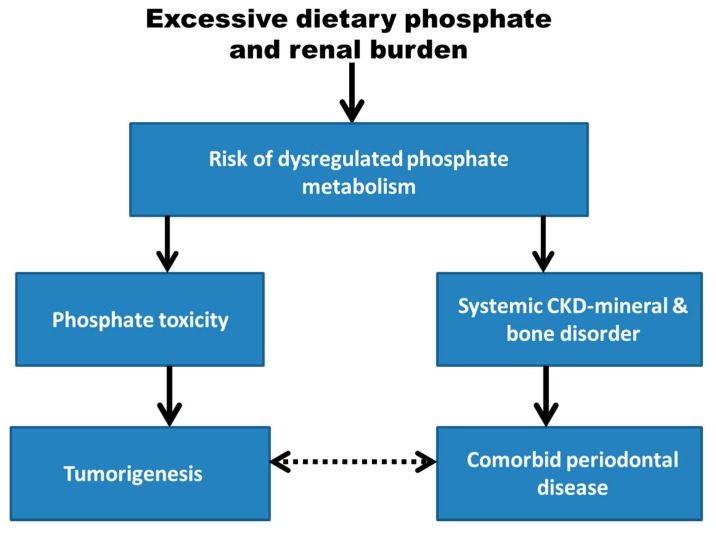
Evidence suggests that excessive dietary phosphate and renal burden increases the risk of dysregulated phosphate metabolism. It is hypothesized that dysregulated phosphate metabolism could act as a mediator or intermediary variable linking phosphate toxicity and systemic chronic kidney disease-mineral and bone disorder (CKD-MBD). Phosphate toxicity may progress to tumorigenesis, and periodontal disease may occur as a comorbidity with systemic CKD-MBD, which forms an association between tumorigenesis and periodontal disease (dotted arrow).

As an alternative explanation for the development of periodontal disease and its association with cancer, Figure 1 in this perspective article shows that excessive dietary phosphate and renal burden may increase the risk for dysregulated phosphate metabolism. It is hypothesized that this systemic metabolic pathology could mediate the association of tumorigenesis with periodontal disease through separate causal pathways involving phosphate toxicity and systemic chronic kidney disease-mineral and bone disorder (CKD-MBD), respectively.

## 5. Chronic Kidney Disease-Mineral and Bone Disorder

Phosphate metabolism is often dysregulated in chronic kidney disease (CKD), and hyperphosphatemia contributes to patient morbidity and mortality [48]. CKD is often comorbid with cancer [49], and an association of periodontal disease with CKD has been confirmed in observational studies [50,51,52]. As in cancer, it is plausible that the association of periodontal disease with CKD also involves dysregulated phosphate and calcium metabolism, seen in systemic CKD-MBD [53]. In addition to endocrine disturbances in the metabolism of ions and hormones associated with bone, characterization of chronic kidney disease-mineral and bone disorder (CKD-MBD) includes abnormalities in bone mineralization, bone growth, and bone turnover. Jaw bones affected with CKD-MBD increase the risk of bone loss in periodontitis [54]. 

While severity of periodontal disease was found to increase with stages of CKD, serum albumin levels declined as periodontal disease severity increased [55]. Of relevance, increasing hyperphosphatemia in CKD is also associated with declining serum albumin levels [56], implying that hyperphosphatemia may also be positively associated with periodontal disease severity. It has been observed that dental calculus formation appears to be similar to ectopic calcification such as that which occurs in kidney stone formation [57], and an association has been found between calculus formation and renal calculi [58]. Of relevance, ectopic calcification, including calcification of the vascular system, is associated with dysregulated phosphate metabolism and hyperphosphatemia [27]. 

Hyperphosphatemia is a common condition in dialysis patients [59], and dialysis patients were found to have a higher rate of dental calculus formation compared to healthy controls [60], further implying a role for high serum phosphate levels in calculus formation. Calculus that formed in closer proximity to salivary gland ducts was also found to contain more calcium and phosphate than calculus in other oral locations [46]. Increased phosphate and calcium in saliva has been associated with higher risk for inflammation of the periodontium [61], and high levels of phosphorus have been linked to systemic inflammation [43], which is often present in periodontal disease and cancer. Of relevance, detection of ribose nucleic acids in saliva is used in oral cancer diagnosis [62], and as previously mentioned, phosphorus is a key element in the formation of nucleic acids detected in circulating microRNA [32]. Furthermore, increased periodontal risk has been associated with carotid artery calcifications [63] and increased aortic arch plaque thickness [64], which may be related to vascular ectopic calcification caused by elevated serum phosphate associated with periodontal disease.

Recent studies confirm an association of systemic osteoporosis with periodontal disease [65,66,67,68,69,70,71,72]. Bone resorption related to dysregulated phosphate metabolism may be initiated when high levels of phosphate in the serum unite with free calcium ions (Ca2+) to form excess levels of calcium phosphate, which induces hypocalcemia. In response, parathyroid hormone secretion resorbs calcium from bone, which immediately restores low serum levels of free calcium [73]. The paradox of normal serum calcium levels in osteoporosis explains why osteoporosis is considered a silent disease, which is often not diagnosed until fractures occur. According to the guidelines of KDIGO (Kidney Disease: Improving Global Outcomes), bone turnover markers like serum levels of PTH are the preferred diagnostic indicator for impaired bone quality, rather than invasive bone biopsies [74]. As excess levels of calcium phosphate rise in the serum, calcium phosphate may be deposited in soft tissue forming ectopic calcifications. Abnormal calcification may also occur in bone itself, as seen in osteoblastic metastasis in breast, prostate, and other bone metastatic cancers [75]. Of relevance, the concurrent systemic pathologies of osteoporosis and ectopic calcification were first described as metastatic calcification by Virchow in 1855 [76]. While conducting autopsies, Virchow observed that calcium phosphate appeared to flow out of the bones and metastasize to other tissue, although he did not understand the mediating role of PTH in this pathology. A similar PTH mechanism associated with dysregulated phosphate (as seen in secondary hyperparathyroidism) could help explain why ectopic dental calculus forms concurrently with alveolar bone resorption and tooth loss in periodontal disease. Further research is needed to examine the role of dysregulated phosphate metabolism and hyperphosphatemia in periodontal disease.

## 6. Conclusions

Explaining a common cause linking periodontal disease with cancer has possible global health implications, suggesting that a reduction in the global burden of these non-communicable diseases may be possible by modifying their common lifestyle risk factors and determinants. This perspective article hypothesizes that the association of cancer with periodontal disease may be mediated by the pathophysiological determinant of dysregulated phosphate metabolism related to high dietary phosphate intake and kidney burden. Evidence supports the role of phosphate toxicity in cancer development, and periodontal disease is an associated comorbidity with chronic kidney disease, in which systemic CKD-mineral and bone disorder is common. More research is needed to establish the role of dysregulated phosphate metabolism in periodontal disease.
